# Dichloroacetate alleviates development of collagen II-induced arthritis in female DBA/1 mice

**DOI:** 10.1186/ar2799

**Published:** 2009-09-01

**Authors:** Li Bian, Elisabet Josefsson, Ing-Marie Jonsson, Margareta Verdrengh, Claes Ohlsson, Maria Bokarewa, Andrej Tarkowski, Mattias Magnusson

**Affiliations:** 1 Department of Rheumatology and Inflammation Research, Sahlgrenska Academy, University of Gothenburg, Guldhedsgatan 10A, Box 480, SE-405 30, Gothenburg, Sweden; 2 Centre for Bone Research, Department of Internal Medicine, Sahlgrenska University Hospital, Gröna Stråket 8, SE-413 45, Gothenburg, Sweden

## Abstract

**Introduction:**

Dichloroacetate (DCA) has been in clinical use for the treatment of lactacidosis and inherited mitochondrial disorders. It has potent anti-tumor effects both *in vivo *and *in vitro*, facilitating apoptosis and inhibiting proliferation. The pro-apoptotic and anti-proliferative properties of DCA prompted us to investigate the effects of this compound in arthritis.

**Methods:**

In the present study, we used DCA to treat murine collagen type II (CII)-induced arthritis (CIA), an experimental model of rheumatoid arthritis. DBA/1 mice were treated with DCA given in drinking water.

**Results:**

Mice treated with DCA displayed much slower onset of CIA and significantly lower severity (*P *< 0.0001) and much lower frequency (36% in DCA group vs. 86% in control group) of arthritis. Also, cartilage and joint destruction was significantly decreased following DCA treatment (*P *= 0.005). Moreover, DCA prevented arthritis-induced cortical bone mineral loss. This clinical picture was also reflected by lower levels of anti-CII antibodies in DCA-treated versus control mice, indicating that DCA affected the humoral response. In contrast, DCA had no effect on T cell- or granulocyte-mediated responses. The beneficial effect of DCA was present in female DBA/1 mice only. This was due in part to the effect of estrogen, since ovariectomized mice did not benefit from DCA treatment to the same extent as sham-operated controls (day 30, 38.7% of ovarectomized mice had arthritis vs. only 3.4% in sham-operated group).

**Conclusion:**

Our results indicate that DCA delays the onset and alleviates the progression of CIA in an estrogen-dependent manner.

## Introduction

The pyruvate dehydrogenase activator dichloroacetate (DCA) is a small molecule that has been used in humans for decades as a treatment for acquired and congenital forms of lactacidosis by shifting pyruvate metabolism from cytoplasmic lactate production to oxidative production of acetyl-CoA in the mitochondria [1]. Most recently, DCA was found to act as an efficient tumor growth inhibitor, both *in vitro *and *in vivo*, by shifting glucose metabolism from glycolysis to glucose oxidation in malignant cells. This shifting results in the release of pro-apoptotic mediators and decreases proliferation in malignant cells, thus eliminating active tumor cells while leaving the normal cells unaffected [1].

Rheumatoid arthritis (RA) is a systemic autoimmune disease characterized by chronic joint inflammation [2]. The prevalence of RA is 0.5% to 1% of the population worldwide. Females have a higher incidence (3:1) than males. Several lines of evidence show that the female hormone estrogen affects both the incidence and the progression of RA in humans [3,4] and in animal models [5,6]. RA is characterized by synovial cell proliferation and infiltration of inflammatory cells to the synovium. Cytokine production by these cells (for example, tumor necrosis factor-alpha [TNF-α] and interleukin [IL]-1, IL-6, and IL-17) plays a pivotal role in RA [7]. These cytokines, notably TNF [8] and IL-6 [9], may promote the development of osteoclasts [10], which increases bone erosion and systemic bone loss [11].

Because the cause of RA is complex and elusive, it continues to present therapeutic challenges, especially erosive arthritis. Murine collagen II (CII)-induced arthritis (CIA) is a widely used experimental model of RA and shares many histopathological features of the human counterpart [12]. It is usually used to investigate mechanisms relevant to RA as well as new anti-arthritic treatments [13]. As in the case of RA, CIA is primarily an autoimmune disease of the joints [14] with increased angiogenesis, inflammatory cell infiltration, synovial hyperplasia, and bone erosion. Because of the anti-proliferative and pro-apoptotic properties of DCA, we hypothesized that DCA may inhibit the development of arthritis in CIA. To this end, DCA was added to drinking water at the time of induction of CIA. Our results suggest that DCA significantly delays the onset and development of destructive arthritis in female DBA/1 mice. The protective effect of DCA was mediated in part via estrogen-dependent pathways.

## Materials and methods

### Mice

DBA/1 mice (Taconic Europe A/S, Ry, Denmark), 6 to 8 weeks old, were used for CIA experiments. For the delayed-type hypersensitivity (DTH) experiment, 6 to 8 week old mice were used. All of the mice were maintained in the animal facility of the Department of Rheumatology and Inflammation Research, University of Gothenburg, Sweden, in accordance with the local ethics board animal husbandry standards. Mice were housed up to 10 animals per cage under standard conditions of light and temperature and fed with standard laboratory chow *ad libitum*.

### Collagen II-induced arthritis

Chicken CII (Sigma-Aldrich, St. Louis, MO, USA) was dissolved at a concentration of 2 mg/ml in 0.1 M acetic acid and then emulsified in an equal volume of complete Freund's adjuvant (Sigma-Aldrich). Arthritis was induced by intradermal injection of DBA/1 mice at the base of the tails with 100 μL of the emulsion. Booster immunization containing 100 μg of CII in incomplete Freund's adjuvant (Sigma-Aldrich) was administered 21 days after the priming. The experiments were terminated in 6 to 8 weeks.

### Dichloroacetate treatment

CIA was used to investigate the effect of DCA (sodium DCA 99% purity; BuyDCA, Sonora, CA, USA) on arthritis. DCA was administered by dissolving it in the drinking water. Control mice were given water only. The average amount of DCA per mouse taken was determined by measuring the volume of DCA solution that mice consumed in each cage. We calculated and adjusted the concentration of DCA required to achieve a daily dose of 0.3 mg or 3 mg DCA/mouse per day. DCA was provided in the drinking water from day 0 of all experiments. The experiment was repeated three times as outlined in Table 1. To study the impact of estrogen on DCA-mediated effects, endogenous estrogen production in female DBA/1 mice was blocked by ovariectomy (OVX). Sham-operated mice were used as controls. DBA/1 mice were regularly weighed from the day of priming and checked for the development of arthritis after booster immunization. When the experiments were terminated, blood was drawn for serological analyses. Paws were processed for histological analyses.

**Table 1:** Distribution of mice in dichloroacetate treatment of collagen II-induced arthritis

	Gender	Treatment	Number of mice	Arthritis index^a^
First experiment	Female	DCA	4	3/4 (0.75)
	Female	Water	4	30/4 (7.5)
	Male	DCA	6	35/6 (5.8)
	Male	Water	5	22/5 (4.4)
Second experiment	Female	DCA	10	6/10 (0.6)
	Female	Water	10	34/10 (3.4)
	Male	DCA	10	31/10 (3.1)
	Male	Water	10	39/10 (3.9)
Third experiment	Female	DCA	8	7/8 (0.9)
	Female	Water	8	38/8 (4.75)
OVX experiment 1	OVX	DCA	10	16/10 (1.6)
	Sham	DCA	10	3/10 (0.3)
OVX experiment 2	OVX	DCA	21	63/21 (3.0)
	Sham	DCA	19	23/19 (1.2)

^a^Arthritis index at termination of experiments. DCA, dichloroacetate; OVX, ovariectomy; Sham, sham-operated.

### Clinical evaluation of arthritis

All of the DBA/1 mice were inspected every second or third day after booster to assess the presence of arthritis. To evaluate the intensity of arthritis, a clinical scoring system of 0 to 3 points for each paw was used: 0, no sign of inflammation; 1, mild swelling or erythema or both; 2, moderate swelling and erythema; and 3, marked swelling and erythema. The arthritic index for each mouse was constructed by summing up the scores of all four limbs.

### Delayed-type hypersensitivity reaction

To assess the impact of DCA on a T cell- and macrophage-dependent inflammatory response [15,16], the DTH reaction was performed. Thirty female mice were divided into three groups (10 mice per group). Two groups were provided DCA in drinking water (0.3 and 3 mg/mouse per day, respectively). The control group was provided water only. After 2 days, all of the mice were immunized by epicutaneous application of 150 μL of a mixture of ethanol acetone (2:1) containing 3% (vol/vol) oxazolone (OXA) (Sigma-Aldrich) on the abdomen skin. One week after the priming, the right ears were challenged on both sides by topical application of 30 μL of 1% OXA, which was dissolved in olive oil. Thirty microliters of olive oil only was applied to the left ears as vehicles. The intensity of DTH reaction was examined as previously described [17].

### Olive oil-induced inflammation

Olive oil-induced skin inflammation is granulocyte-mediated but T cell- and monocyte-independent [18]. A single intradermal injection of olive oil into mouse footpad induces massive infiltration of polymorphonuclear cells, which give rise to a localized footpad swelling. The thickness of footpad can be measured and relates to severity of the inflammatory process [19]. Thirty microliters of olive oil (Apoteksbolaget, Göteborg, Sweden) was injected intradermally in a hind foot dorsum of mice. Footpads were measured before and 24 hours after injection using an Oditest spring caliper (Kröplin, Schluchtern, FRG). The footpad swelling was expressed as footpad increased thickness (in millimeters) after injection and was scored as described previously [20].

### Analyses of hormone, antibody, and cytokine levels

The level of serum hormones was analyzed by the following radioimmunoassays: insulin-like growth factor (IGF1) (Mediagnost, Reutlingen, Germany), testosterone (MP Biomedicals, Irvine, CA, USA), and cortisol (CIS Bio International, Marcoule, France). Uteri were weighed as an indirect indicator of estradiol level [21]. Anti-CII antibody analyses were performed as previously described [22]. IL-6 levels in sera were analyzed as described before [23].

### Histological examination

All four paws from DBA/1 mice were excised, followed by routine fixation, decalcification, and paraffin embedding. Tissue sections were stained with hematoxylin/eosin. The sections were studied by a blinded examiner regarding synovitis and erosion of bone/cartilage. Synovial hypertrophy was defined as a membrane thickness of more than two cell layers [23]. A histological scoring system was used: 1, mild; 2, moderate; and 3, severe synovitis or bone erosion [24]. Knee joints, ankles, toes, elbows, wrists, and hands were examined. A mean score from all of the inspected paws for each animal was calculated [25].

### Impact of dichloroacetate on bone mineral density

The left femurs from DBA/1 mice were fixed in 4% (vol/vol) buffered formaldehyde for 3 days and then replaced by 70% (vol/vol) alcohol until analyses of bone mineral density (BMD) were performed. A peripheral quantitative computed tomography (pQCT) scan with a Stratec pQCT XCT Research M (Norland, Fort Atkinson, WI, USA) was used as previously described [26]. Trabecular BMD was analyzed with a metaphyseal scan at a point located at a distance of 3% of the length of the femur from the distal growth plate. The inner 45% of the area was defined as the trabecular bone compartment. Cortical bone parameters were determined with a middiaphyseal scan, which contained only cortical bone.

### Statistical analysis

Statistical analyses were performed by using the Mann-Whitney *U *test and the chi-square test. Values are reported as median ± 10% to 90% range. A *P *value of less than 0.05 was considered significant.

## Results

### Effect of dichloroacetate on development of collagen II-induced arthritis

**Figure 1:**
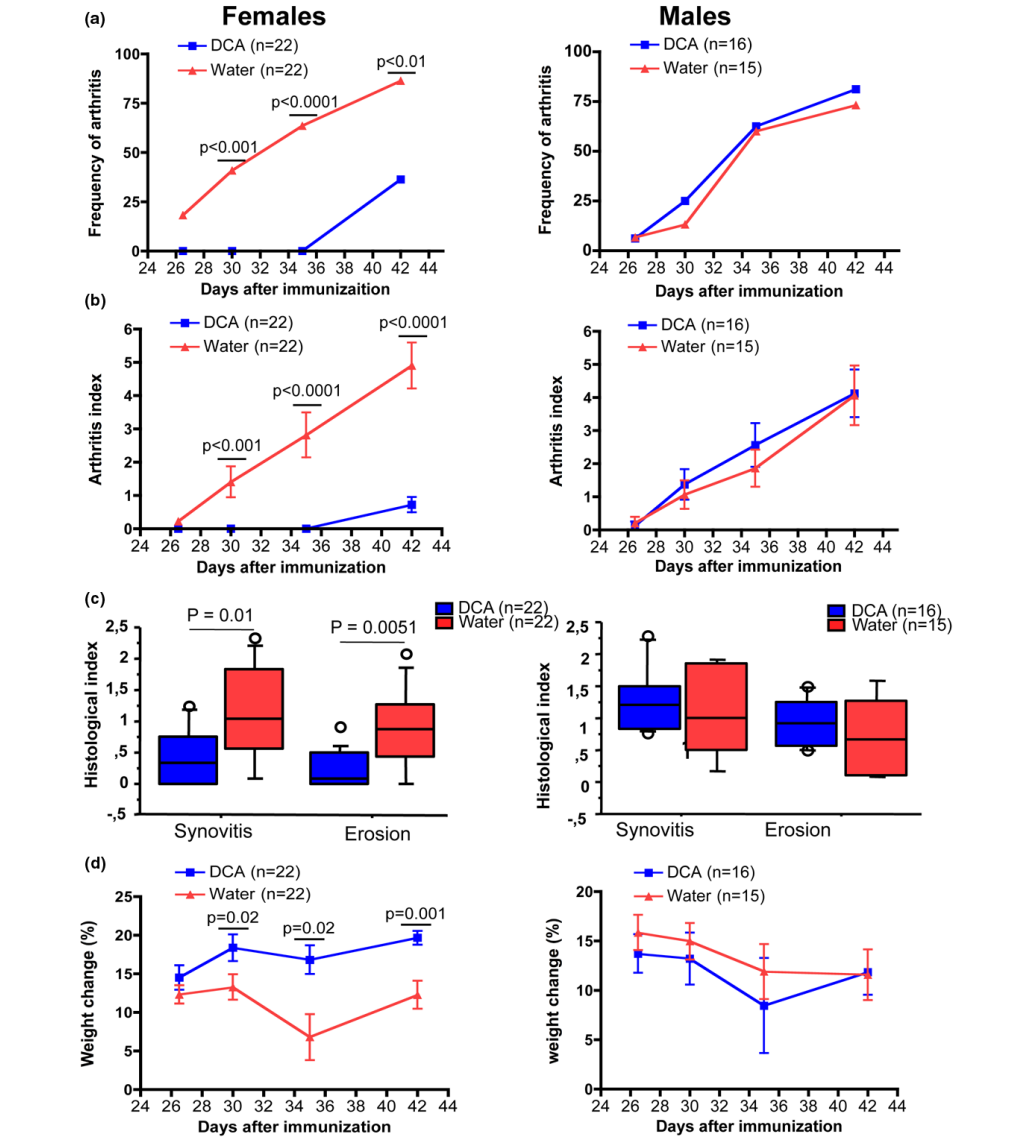
Frequency, severity of arthritis, bone destruction, and weight change in collagen II (CII)-induced arthritis mice treated with dichloroacetate (DCA) (3 mg/mouse per day) or water. **(a) **DCA effect on frequency of arthritis in both female and male CII arthritis mice. **(b) **DCA treatment on both female and male mice in regard to severity of arthritis. **(c) **Synovitis and bone erosion in CII-immunized DBA/1 with or without DCA treatment. **(d) **Weight change in DCA-treated mice and water controls during the course of CII-induced arthritis experiments. Values from three independent experiments were pooled. The DCA-treated group and the water-drinking group each contained 22 mice.

To evaluate whether DCA had an effect on the development of CIA, male and female DBA/1 mice were provided drinking water with or without DCA from the priming day until the experiment was terminated. At a dose of 3 mg DCA/mouse per day, for female DBA/1 mice, none of the 22 mice that drank DCA had signs of arthritis 37 days after the priming with CII, whereas most of the control mice (16 of 22, or 73%) (*P *< 0.0001) already had ongoing arthritis (Figure 1a). Female mice that drank DCA had a much lower severity of arthritis (Figure 1b). For male mice, there was no difference between the DCA-drinking group and the water group in regard to the onset of arthritis and its severity. In the dose of 0.3 mg DCA/mouse per day, we did not find any impact of DCA on the development and course of CIA (data not shown).

**Figure 2:**
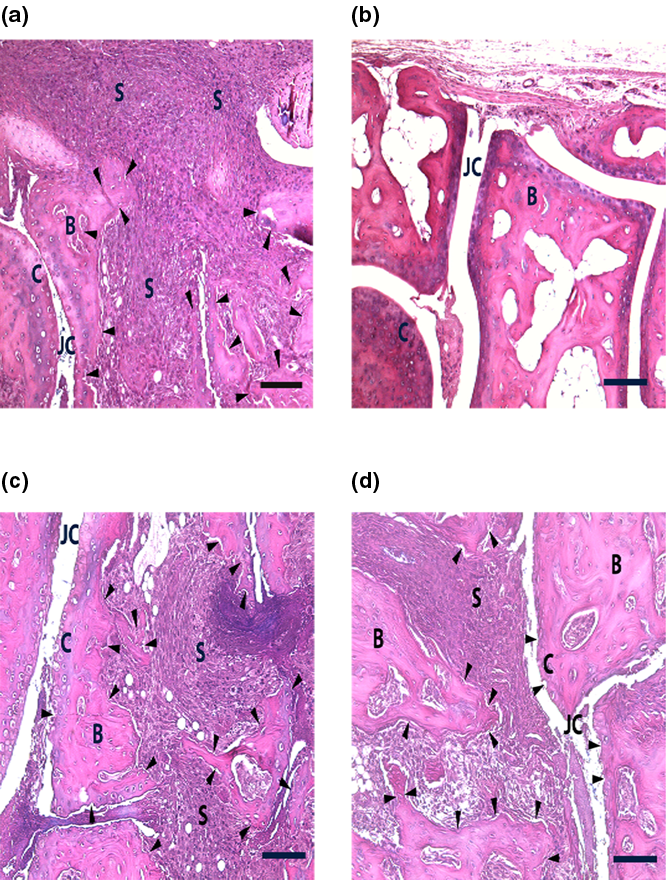
Micrographs of tarsal joints from collagen II-immunized female and male DBA/1 mice. **(a) **Representative image of a tarsal joint from a female water-drinking mouse. **(b) **Representative image of a tarsal joint from a female dichloroacetate (DCA)-drinking mouse (3 mg DCA/mouse per day). **(c) **Representative image of a tarsal joint from a male water-drinking mouse. **(d) **Representative image of a tarsal joint from a male DCA-drinking mouse (3 mg DCA/day). Arrowheads indicate erosion of bone and cartilage. Scale bar = 100 μm. B: bone; C: cartilage; JC: joint cavity; S: synovitis.

Histological sections from female mice confirmed that the DCA group had a lower severity of arthritis. Notably, the destruction of bone and cartilage was significantly diminished in the DCA-drinking group compared with the control group (Figures 1c and 2). In contrast, there was no significant difference in male mice between the two groups. Importantly, DCA-treated mice did not show decreased weight gain as compared with control mice (Figure 1d), indicating that DCA was not toxic. In fact, female mice receiving DCA gained slightly more weight than their controls.

### Effect of dichloroacetate on inflammatory immune responses

**Figure 3:**
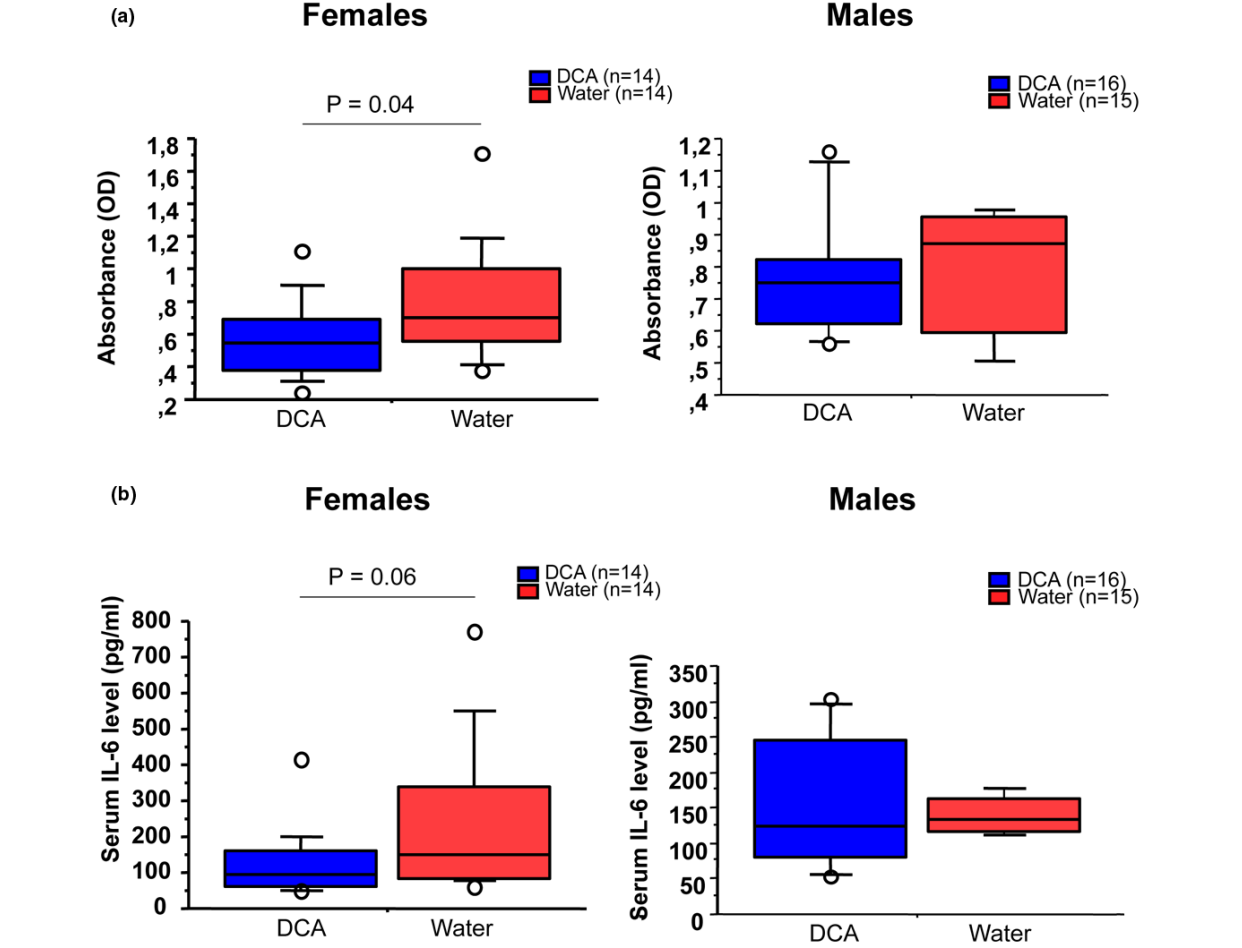
Dichloroacetate (DCA) impact on *in vivo *inflammatory immune response. **(a) **IgG anti-collagen II antibody levels from the DCA-drinking group and the water-drinking group. **(b) **Serum pro-inflammatory cytokine intereukin-6 (IL-6) levels from DCA-drinking mice and water controls. Values from two experiments were pooled. The DCA-drinking group and the water-drinking group each contained 14 mice. OD: optical density.

A potential mechanism by which DCA could suppress CIA is the blocking of anti-CII antibody production. Anti-CII antibody levels and serum IL-6 were analyzed in serum obtained at the termination of experiments. In female mice, but not in male mice, circulating anti-CII IgG antibody levels were significantly decreased in the DCA-drinking group compared with the water group (*P *= 0.04) (Figure 3a). The serum IL-6 level in the DCA-drinking group was lower than in the water group, but the data did not reach statistical significance (*P *= 0.06) (Figure 3b). Thus, the anti-inflammatory effect of DCA on arthritis is accompanied by lower levels of anti-CII antibodies.

### Impact of dichloroacetate on in vivo cell-mediated inflammatory responses

DTH is a T cell-mediated immune reaction. To test the effect of DCA on DTH, DCA-treated mice and water controls were epicutaneously immunized and challenged with OXA. DTH reactivity was registered by measuring the increase in ear thickness 24 hours after the challenge. We did not find any significant differences in regard to the severity of DTH between DCA-treated mice and their controls (data not shown). Likewise, DCA had no effect on the granulocyte-dependent olive oil-induced inflammation (data not shown).

### Dichloroacetate treatment ameliorates the cortical bone loss induced by arthritis

**Figure 4:**
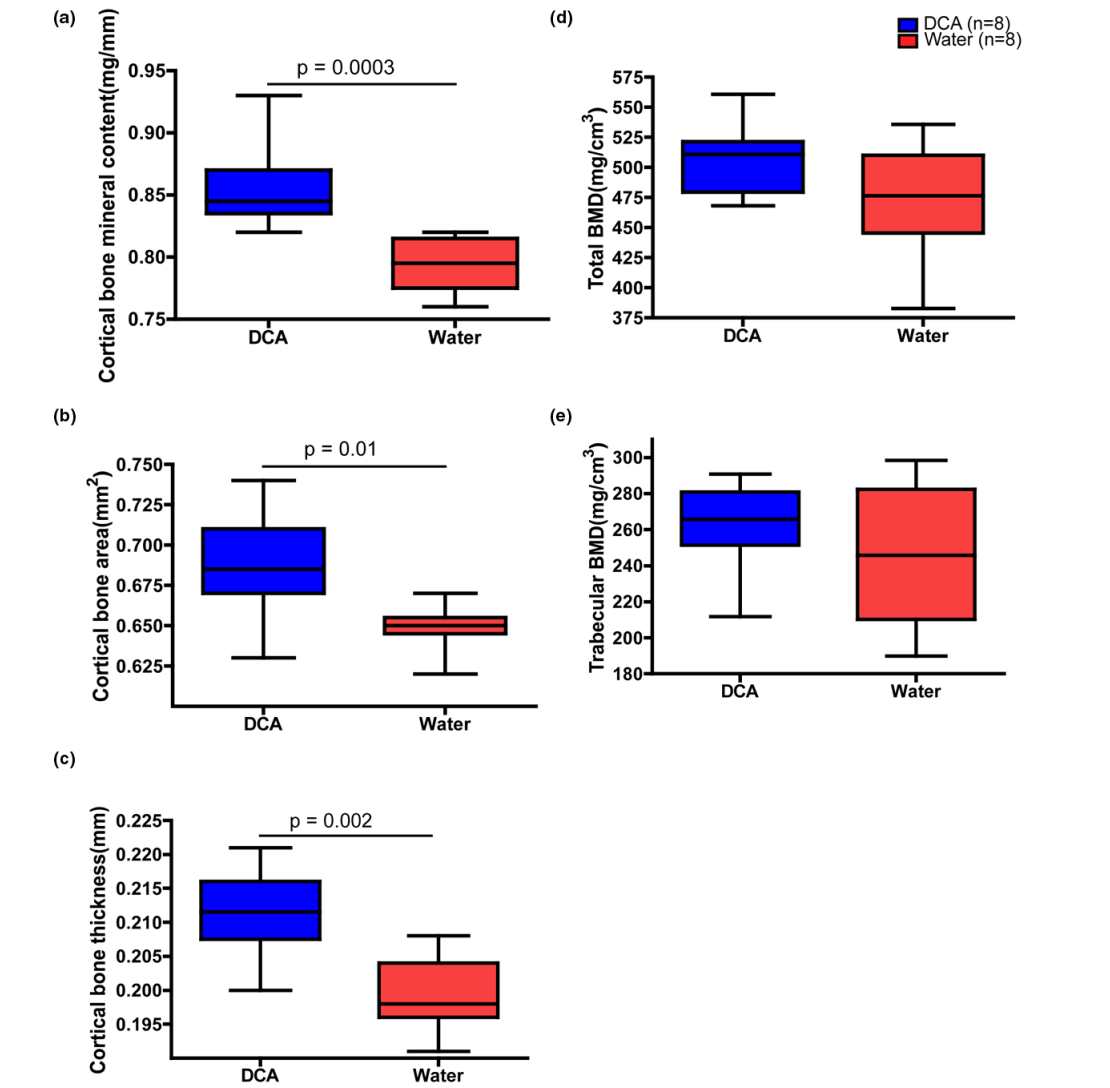
Dichloroacetate (DCA) effect on bone mineral density (BMD) (female mice only). **(a) **Cortical bone mineral content in mice treated with DCA or water. **(b) **The thickness of cortical bone in the DCA-drinking group and the water-drinking group. **(c) **Cortical bone area in the DCA-drinking group and the water-drinking group. **(d) **Total BMD measured in the DCA group and water controls. **(e) **Trabecular BMD in the DCA-treated group and water controls. One experiment was performed with eight female mice in the DCA-drinking group and eight female mice in the control group.

To evaluate whether the ameliorative effect of DCA on CIA also was reflected in the protection of arthritis-induced bone loss, the left femur of each mouse was subjected to a pQCT scan at the termination of CIA. Mice immunized with CII and treated with DCA displayed significantly higher cortical bone mineral content than did the water-drinking group (*P *= 0.001) (Figure 4a). Likewise, thickness of cortical bone (*P *= 0.039) (Figure 4b) and cortical bone area (*P *= 0.01) (Figure 4c) were significantly higher in the DCA-treated group. No differences were observed with respect to total BMD and trabecular BMD between the DCA-drinking group and their water controls (Figure 4d, e).

### Impact of ovariectomy on dichloroacetate treatment of arthritis

**Figure 5:**
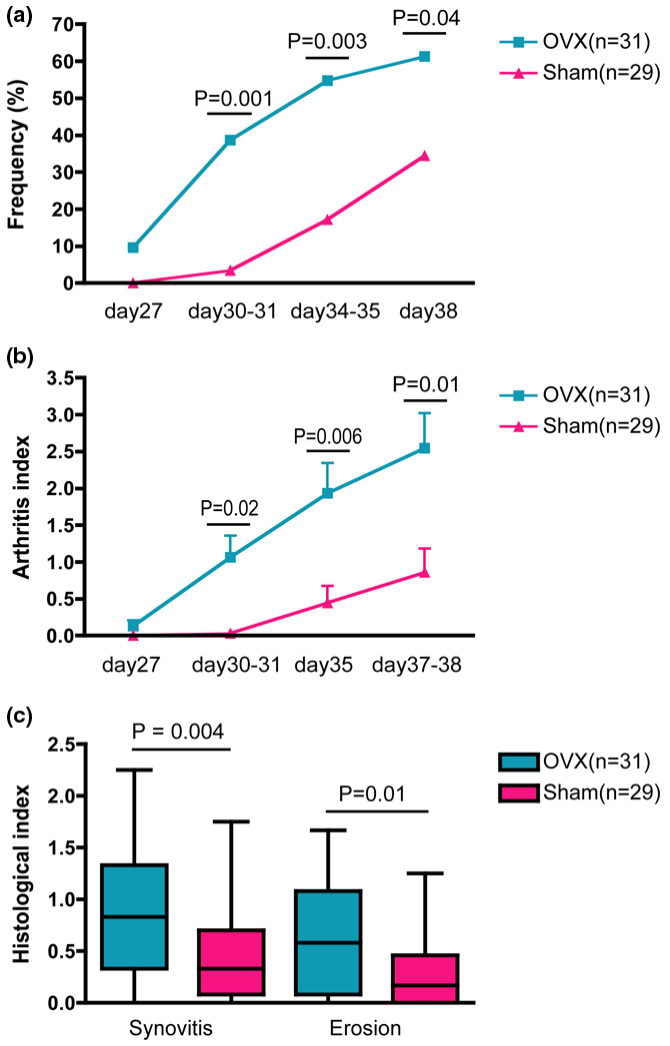
Impact of ovariectomy (OVX) on dichloroacetate (DCA) treatment of arthritis. Frequency **(a) **and severity **(b) **of arthritis in DCA-treated ovariectomized mice and in sham-operated mice treated with DCA. **(c) **Histological index of OVX and sham-operated mice treated with DCA. Results of two experiments were pooled with 31 OVX mice and 29 sham-operated mice.

Because only female mice responded to DCA therapy, we hypothesized that the beneficial effect of DCA on arthritis was related to estrogen. To evaluate the importance of estrogens, one group of DBA/1 mice was ovariectomized (OVX) and another group had sham surgery. After 1 week, both groups were primed and booster - immunized with CII and treated with DCA (3 mg DCA/mouse per day) as previously described. On day 30, 12 of 31 mice in the OVX group had signs of arthritis (38.7%) compared with only 1 of 29 mice in the sham controls (3.4%) (*P *= 0.001). On day 34, 17 of 31 in the OVX group had arthritis (54.8%) but only 5 of 29 in the sham-operated group did (17.2%) (*P *= 0.003). When the experiment was terminated on day 38, 19 of 31 mice in the OVX group had developed arthritis (61.3%). In contrast, only 10 of 29 mice in the sham-operated group (34.5%) had signs of arthritis (*P *= 0.04) (Figure 5a). The sham-operated group also had much less severe arthritis than the OVX group (*P *= 0.006) (Figure 5b). Histological analysis showed that the OVX group had synovitis (*P *= 0.004) and bone erosion (*P *= 0.01) that were significantly more severe compared with sham-operated controls (Figure 5c). But no difference was found between the OVX group and the sham-operated group in regard to weight changes (data not shown).

Although OVX mice and sham-operated mice had different arthritis responses following DCA treatment, we did not find any difference between the OVX group and the sham group in regard to level of IL-6 (OVX: median 76 pg/mL, range 20 to 1,032 pg/mL versus sham: median 111.2 pg/mL, range 30 to 348.2 pg/mL) and anti-CII antibodies (optical density: OVX: median 0.91, range 0.37 to 1.32 versus sham: median 0.84, range 0.30 to 1.69). As an indirect indicator of estrogen levels, the weight of uteri from DCA- and water-treated intact female mice was recorded. No difference was found between the DCA group and their controls (DCA: median 50 mg, range 26 to 111 mg, n = 8 versus water: median 49 mg, range 15 to 104 mg, n = 8). We also measured the level of testosterone. There was no significant difference between the DCA group and the control group (Data not shown).

## Discussion

DCA delays the onset of CIA and at the same time alleviates the progress of CIA. Interestingly, this outcome is observed in female mice only. Female DBA/1 mice treated with DCA had a significant slower onset and less severe arthritis compared with water-treated controls. Importantly, the destructive action of inflammation on bone was almost totally inhibited in mice provided with DCA. The results of this study reveal for the first time that destructive arthritis can be inhibited by DCA administration. Our results also suggest that estrogen plays an important role in the beneficial effect of DCA.

DCA is a well-established drug used for the treatment of lactic acidosis. It also exhibits efficient anti-tumor properties due to its pro-apoptotic and anti-proliferative effects without visibly affecting non-cancerous cells or eliciting systemic toxicity [1]. RA, like a malignant tumor, is also characterized by increased cell proliferation. We therefore wanted to evaluate whether DCA could prevent the development of arthritis in a model of RA. To this end, we treated CIA in DBA/1 mice with DCA. We found that DCA can ameliorate arthritis potently, but only in female mice.

How does DCA abolish the development of chronic destructive inflammation in CIA? B cells are important in the pathogenesis of RA by producing auto-antibodies and in T-cell activation [27]. Antibodies to CII have been detected in serum and synovial fluid of patients with RA [28,29]. As in RA, anti-CII antibody production contributes to the development of CIA [30,31]. The beneficial effect of DCA on arthritis is likely due to the down-regulation of B cells producing anti-CII antibodies because DCA-treated mice displayed significantly lower levels of anti-CII antibodies (Figure 3a). This may be a direct effect of DCA or mediated via reduction of pro-inflammatory cytokines. IL-6 is a pro-arthritogenic cytokine that affects B cells by promoting plasma cell differentiation, antibody production, and class switch [32-34]. A tendency toward lower serum levels of IL-6 was observed in DCA-treated female mice compared with water-treated mice (Figure 3b), which thus reflects a possible role of IL-6 in DCA-mediated inhibition of anti-CII antibody production. We could not show any effect of DCA on T cell-mediated inflammation (DTH) or granulocyte-mediated inflammation (olive oil-induced), suggesting that the observed beneficial effect is through humoral immunity, as indicated by the effect on anti-CII antibody production.

Because only female DBA/1 mice displayed amelioration of arthritis in response to DCA treatment, female hormones such as estrogen may play a role. A role of estrogens in RA is suggested by the therapeutic effect of estradiol in menopausal women with RA, a group of patients characterized by low levels of estrogens and high incidence of RA [3,5,35,36]. In addition, estradiol has both prophylactic and therapeutic effects on arthritis development in CIA [37]. To test the hypothesis, endogenous estrogens were removed by OVX. The OVX group treated with DCA had an earlier onset of disease, more frequent and more severe arthritis, and more synovitis and bone destruction compared with the sham-operated group treated with DCA, which had intact estrogen production. A confounding factor is the fact that the OVX treatment in itself may worsen CIA [36], which could possibly mask anti-inflammatory effects of DCA. But the net effect of DCA on sham versus OVX mice is of several magnitudes greater than the effect of OVX on arthritis. DCA ameliorates arthritis by decreasing the frequency of arthritis by at least 40% in sham versus OVX mice (Figure 5a, day 38) and the severity by at least 65% (Figure 5b, days 37 and 38), whereas the earlier reported aggravating effect of OVX on arthritis severity is 20% and the effect on arthritis frequency is minimal or non-existent [36]. This suggests that the observed difference between mice unable to produce estrogens and water controls is indeed due to the DCA treatment. An ameliorating effect of estrogens on arthritis has been demonstrated in both mice and humans [3,5,35,36]. However, the effect of DCA is probably not via increased production of estrogens as DCA treatment did not cause increased uterus weight. Rather, DCA elicits its effect by affecting estrogen signaling.

The beneficial effect of DCA on CIA may not be entirely dependent on estrogens. This is supported by the fact that DCA-treated mice unable to produce estrogen (OVX mice) had a lower frequency of arthritis (61.3%) than water-treated mice (86.4%) (Figures 5a and 1a, respectively). As only female mice benefited from DCA treatment, this observation indicates that DCA has some estrogen-independent but gender-dependent effects on inflammation. Also, the difference in response to DCA could be due to the fact that male hormones may inhibit the DCA effect.

Previous studies of DCA in inflammation have not focused on the effect on bone density. Our results clearly indicate that DCA can prevent cortical bone mineral loss in female mice in CIA (Figure 4a) as a result of increased cortical thickness (Figure 4b). This is in line with a recent study showing that down-regulation of arthritis severity will lead to not only absence of local erosion (that is, in cartilage and subchondral bone) but also systemic effects on BMD [36]. We believe that the beneficial effect of DCA on bone is mediated by a combination of estrogen-dependent effects and the decrease of the inflammatory response manifested by a reduced level of anti-CII antibodies. First, estrogen is important for bone maintenance and may provide protection from bone destruction in arthritis [3,5,35,36]. The role of estrogen in the DCA-mediated effect was demonstrated by the fact that female mice, but not male or OVX-treated female mice, benefited from DCA treatment. Second, the lower levels of anti-CII antibodies found in DCA-treated animals may also prevent bone destruction as anti-CII antibodies induce bone erosions in the CIA model [38]. Such bone erosions may be mediated via anti-CII antibody-dependent C3 recruitment to the cartilage surface, which will initiate an immunological attack, eventually leading to bone destruction [39]. The DCA-mediated reduction of the pro-inflammatory cytokine IL-6 may also contribute to bone protection as IL-6 has potent effects on cartilage and bone destruction [40].

The effect of DCA on cortical bone may also be indirect. As animals have less arthritis, they are more prone to physical activity, which may account for the difference in cortical BMD [41] between treated and non-treated animals (Figure 4). This notion is furthermore in line with the observation that the effect of DCA on bone was mainly on cortical bone, and not on trabecular bone, which is less affected by physical training [42].

## Conclusions

Here, we show for the first time that DCA, a potent drug against lactacidosis, also can protect against the development of arthritis in female mice. DCA ameliorates the development of destructive arthritis in part via estrogen and in part via direct down-modulation of inflammation. This warrants future studies of the therapeutical effect of DCA on already established arthritis. The recent long-term clinical trial of oral DCA in children showed that DCA is well tolerated and safe [43], suggesting that DCA can be a potential tool for treating female patients with RA.

## Abbreviations

BMD: bone mineral density; CII: collagen II; CIA: collagen II-induced arthritis; DCA: dichloroacetate; DTH: delayed-type hypersensitivity; IL: interleukin; OVX: ovariectomy; OXA: oxazolone; pQCT: peripheral quantitative computed tomography; RA: rheumatoid arthritis; TNF: tumor necrosis factor.

## Competing interests

The authors declare that they have no competing interests.

## Authors' contributions

LB helped carry out all of the *in vivo *and *in vitro *experiment procedures and statistical analysis and contributed to the experimental design, critical evaluation of the results, and preparation of the manuscript. EJ, I-MJ, and MV helped carry out all of the *in vivo *and *in vitro *experiment procedures. AT contributed to the experimental design, critical evaluation of results and preparation of the manuscript. CO contributed to the experimental design, critical evaluation of the results, and preparation of the manuscript. MM and MB contributed to critical evaluation of the results, statistical analysis and preparation of the manuscript. All authors read and approved the final manuscript.
